# Electrospun PCL Wires Loaded with Vancomycin on Zirconium Substrate

**DOI:** 10.3390/ma16227237

**Published:** 2023-11-20

**Authors:** Ramona-Daniela Radu (Dusman), Manuela Elena Voicu, Mariana Prodana, Ioana Demetrescu, Valentina Anuta, Doina Draganescu

**Affiliations:** 1 Department of General Chemistry, Faculty of Chemical Engineering and Biotechnologies, National University of Science and Technology POLITEHNICA Bucharest, 313 Splaiul Independentei, 060042 Bucharest, Romania; dusman.ramona@yahoo.com (R.-D.R.); manuela_elena.voicu@upb.ro (M.E.V.); ioana.demetrescu@upb.ro (I.D.); 2 Academy of Romanian Scientists, 3 Ilfov Street, 050044 Bucharest, Romania; 3 Department of Physical and Colloidal Chemistry, “Carol Davila” University of Medicine and Pharmacy, 020956 Bucharest, Romania; valentina.anuta@umfcd.ro; 4 Department of Pharmaceutical Physics and Informatics, “Carol Davila” University of Medicine and Pharmacy, 020956 Bucharest, Romania; doina.draganescu@umfcd.ro

**Keywords:** Zr, vancomycin, PCL, electrochemical properties, drug release, kinetic models

## Abstract

The current study presents research about electrodeposition in relation to electrospinning PCL wires on a Zr substrate and loading the coating with vancomycin. The structural composition of the coatings was investigated via FT-IR analysis. The morphology evaluated using scanning electron microscopy coupled with energy-dispersive X-ray spectroscopy, for the composition (SEM-EDS), evidenced the presence of the polymer wires, with and without drug vancomycin loading. The wettability of the coatings was evaluated from the hydrophobic–hydrophilic point of view, and the characterization was completed with mechanical and electrochemical tests. All the electrochemical tests performed in simulated body fluid highlighted that PCL represents a barrier against corrosion processes. The quantitative method to evaluate the loading efficiency shows that almost 80% of the total loaded vancomycin is released within 144 h; after the initial burst at 24 h, a steady release of vancomycin is observed over 7 days. A kinetic model of the drug release was also constructed.

## 1. Introduction

Zirconium (Zr) is one of the more interesting elements to use for biomedical applications, along with its alloys, due to its characteristics, such as the ability to form structures similar to hydroxyapatite in simulated body fluids (SBFs) and the good mechanical properties, biocompatibility and corrosion resistance [1,2]. Zr has physical–chemical properties similar to those of titanium (Ti), such as a good corrosion resistance in alkaline, acid and neutral solutions [3]. Zr poses an allotropic transformation like Ti, and it is presumed that phase stability is similar to titanium alloys; the α and β phases appear in the structure [4,5,6,7,8]. Zr forms oxides, ZrO_2_, and is chemically inert, zirconia being biocompatible and well suited to bio-applications [9,10,11]. The anodized Zr structure and nanostructure were especially investigated, being able to incorporate and release drugs usefully nowadays in a time with aggressive bacteria to enhance the antibacterial effects [12,13]. Both in vitro and in vivo investigations have demonstrated excellent biocompatibility, with no notable adverse effects observed and good mechanical properties [14,15,16,17,18,19,20]. Due to the fact that metallic ions appear in the body when mechanical alloy implants are used, there are ways to increase the performance for bio-applications, using some types of surface modifications, leading to the various coatings that exist in the literature [9].

Many polymers that are biodegradable, like poly (L-lactic acid) (PLA) [21], poly (Lactic-co-glycolic acid) (PLGA) [22] and poly(caprolactone) (PCL) [23], are intensively used in bone tissue applications [24,25]. PCL is a semicrystalline biopolymer that has good mechanical resistance when it is used in surgical operations and is environmentally friendly, not producing local acidosis in contact with the surrounding tissue [26]. PCL is also biodegradable and has been extensively used in the biomedical field due to its biocompatibility and tunable degradation rate. Nanowires made from PCL can provide a high surface area-to-volume ratio, making them suitable for drug loading and delivery [8,27]. Nanocoatings, such as polymeric nanowires, offer the advantage of loading drugs, like gentamicin, ibuprofen, cysteine and vancomycin, for implant stabilization within the human body, as well as stabilizing potentially carcinogenic ions from the metallic implants [28].

Vancomycin is a glycopeptide not related structurally to other intensively used antibiotics. Being known for more than 60 years, its antibacterial mechanism was proposed to inhibit the second stage of cell wall synthesis and altering the permeability of the cell membrane [29]. Vancomycin is a potent antibiotic used to treat various bacterial infections, including those caused by methicillin-resistant Staphylococcus aureus (MRSA) [30,31,32,33], and such an ability is extremely important when such bacteria are very aggressive [34]. By loading vancomycin within the PCL nanowires, controlled and sustained drug release can be achieved, improving the effectiveness of the antibiotic and reducing the need for frequent dosing [35,36,37,38]. Zr coated with PCL nanowires embedded with vancomycin can be utilized to develop antimicrobial-coated medical implants [39]. For instance, orthopedic implants, such as joint prostheses or bone screws, may be coated with this material to prevent post-operative infections and improve implant success rates [14,40,41]. PCL nanowires loaded with vancomycin can be incorporated into wound dressings to provide localized and sustained delivery of the antibiotic to the wound site, enhancing the treatment of infected wounds [42,43]. The PCL nanowires loaded with vancomycin can be engineered into drug delivery systems. These systems may be implanted or injected into the body to deliver vancomycin to specific infection sites, providing targeted therapy while minimizing systemic side effects [31,36,37]. Zr coated with PCL nanowires embedded with vancomycin could be used in dental implants or other oral devices to prevent infections and promote successful integration with the surrounding tissues [41,44,45]. It is important to note that the development and implementation of such bio-applications require rigorous testing and validation to ensure safety and efficacy. In the literature, the drug distribution on coated titanium was investigated using FTIR mapping [46,47]. Zirconium oxide at the micro- and nano-levels was incorporated into tissue engineering scaffolds, to prevent bacterial contamination during tissue regeneration [48,49,50]. Vancomycin embedded on Zr with surface modification and various polymer coatings has not been investigated so far, with the literature mentioning loading of this drug only on titanium [47]. These scaffolds provide mechanical support to growing tissues while simultaneously delivering vancomycin to prevent bacterial contamination during tissue regeneration [48,49,50].

Our study being based on PCL electrospun wires on the Zr surface followed by embedding vancomycin into PCL wires has novelty and whole characterization, including that the kinetics of drug release is new and original. The structure and the morphologies of the coatings were studied using Fourier-transformed infrared spectroscopy (FT-IR) and scanning electron microscopy (SEM) coupled with energy-dispersive spectra (EDS) measurements. The length and thickness of the polymeric wires were measured using a function that is included in the SEM software, named SDS-2019. The wettability phenomena were considered through biocompatibility studies, and mechanical properties were observed by contact angle analysis (CA) and microhardness study. The electrochemistry studies were performed using a potentiostat to determine the stability of the coatings and the corrosion parameters.

The drug release study was conducted using UV-VIS spectrometry and HPLC. A kinetic study of the release of vancomycin was performed using five kinetical models, such as Zero Order, First Order, Higuchi, Hixson–Crowell and Korsmeyer–Peppas. Diffusion–dissolution models treat the problems in terms of a hydrophobic polymeric-controlled dissolution and solution-controlled dissolution [51,52]. Being based on PCL electrospun wire deposition on the Zr surface followed by embedding vancomycin into PCL nanowires, the aim of the present study presents novelty and whole characterization, including that the kinetics of drug release is new and original.

## 2. Materials and Methods

### 2.1. Reagents

The materials used were zirconium (Zr) and polycaprolactone granules (PCL), purchased from Sigma Aldrich, USA, chloroform (CHCl_3_) from Carl Roth, Germany, and N,N-dimethylformamide (DMF) from Alfa-Aesar, USA. Simulated body fluid (SBF) with a pH of 7.4 was prepared with salts from Sigma Aldrich (analytical reagent grade) and ultrapure water (Millipore Direct Q 3UV from Merck, Molsheim, France, 18.2 MΩcm^−1^). Vancomycin hydrochloride (C_66_H_75_Cl_2_N_9_O_24_·HCl, 94.3% purity) supplied by Guinama (Valencia, Spain) was dissolved in a solution of simulated body fluid (SBF) and used as a drug.

### 2.2. Equipments and Techniques

A high-power source (PS/EJ30P20, Glassman High Voltage, Inc., High Bridge, NJ, USA) and a pump (Legato 180, KD Scientific, Holliston, MA, USA) were utilized for the electrospinning process.

Fourier-transform infrared spectroscopy (FT-IR, Perkin-Elmer Spectrum 100, Perkin-Elmer, Shelton, CT, USA) analysis was performed in transmittance mode, from 500 to 4000 cm^−1^, to identify the chemical structure of the coated samples. This domain was selected because it is almost the same domain used for vancomycin FTIR analysis in transmittance mode in a recent paper [53].

SEM micrographs were obtained using a Hitachi SU 8230 scanning electron microscope equipped with EDX Oxford detector analyzer (HITACHI High-Technologies Corp., Tokyo, Japan). The samples were placed on a conductive copper sticker and directly analyzed. The sample was observed under low-vacuum mode, using an accelerating voltage of 10 kV and a secondary electron detector.

A contact angle meter (KSV Instruments CAM100, Espoo, Finland) was used to determine the wettability surface of the samples by dripping a 5 μL of SBF solution on three different areas of the samples, and the average was calculated.

Vickers microhardness was determined using the Tukon 1102 microdurimeter (Berg Engineering, Rolling Meadows, IL, USA). For each Zr sample, three measurements were taken in different areas of the sample, and the average value was calculated.

The electrochemical tests were performed in time using a potentiostat-galvanostat AutoLab PGSTAT100N (Metrohm Autolab Instruments, Utrecht, The Netherlands) in an electrochemical cell with SBF solution as the electrolyte, uncoated Zr and coated samples as working electrode, a Pt bare used as counter electrode and a Ag/AgCl as a reference electrode. The working electrode surface was 0.2827 mm^2^. Tafel potentiodynamic and impedance spectroscopy (EIS) studies were performed vs. open circuit potential (OCP).

A UV spectrophotometer model UV1720 (Uvison Technologies Ltd., Kent, UK) was used for evaluation of vancomycin drug loading.

In vitro release tests were performed on a Vision G2 Classic 6 Dissolution Tester (Teledyne Hanson, Chatsworth, CA, USA), using small-volume flat-bottom 150 mL vessels and mini-paddle assembly at 50 rpm. Quantitative determination of the released vancomycin was performed on a Jasco LC-4000 Series HPLC system (JASCO Corporation, Tokyo, Japan).

### 2.3. Substrate Coating Protocol

#### 2.3.1. Deposition of PCL Fibers on Zr Samples

Zr samples with dimensions of 25 mm × 25 mm × 1 mm were polished with SiC paper with increasing grits (P320–P1200) and then cleaned in an ultrasonic bath with distilled water, ethylic alcohol and acetone (10 min each).

The PCL solution was obtained as follows: 0.205 g granules of PCL was dissolved in 1.8 mL CHCl_3_ and 0.2 mL DMF under magnetic stirring for 15 min and then ultrasonication for 10 min for homogenization.

Zr samples were modified with PCL fibers by a mixture of PCL solution, which was transferred in a 1 mL plastic syringe with a needle placed in a pumping system with a constant flow rate of 0.5 mL/h. The pump was connected to a high-voltage power source with a 15 kV constant voltage. The Zr samples were fixed with a double adhesive to a copper collector plate covered with aluminium foil at a distance of 10 cm from the needle tip. The time deposition was 35 min.

#### 2.3.2. Preparation of SBF Solution

The SBF solution was prepared according to our previous paper [54] and was used to characterize the uncoated Zr and coated samples. The solution was prepared adjusting the pH with hydrogen chloride.

### 2.4. Drug Loading

Being one of the first drugs to struggle against MRSA infection, which is well known as a strong threat to public health, vancomycin drug loading and release were studied in various forms. In this study, it was used as a hydrophilic powder, with good solubility. The preparation of the solution consisted of immersing the Zr-PCL nanofiber samples in 10 mL SBF solution containing 2 g/L vancomycin and keeping it for 24 h, followed by calculation of the loading efficiency (Equation (1)).

(1) $$LE\%=\frac{The\;amount\;of\;Vancomycin\;loaded}{Theorical\;Vancomycin\;amount\;in\;the\;PCL\;nanofibers}\cdot 100$$

The release of the vancomycin from the PCL fiber coating was performed by immersion of the coated plates in 10 mL SBF; 0.2 mL samples was withdrawn at predefined intervals in timeframe 0–24 h, and the same volume of fresh SBF was added in order to keep the volume constant. Quantification of vancomycin in the collected samples was performed via UV spectrometry, at 280 nm [55].

### 2.5. Drug Release

The release of the vancomycin from the PCL fiber coating was performed by immersion of the coated plates immobilized on aluminum stubs on the bottom of flat small-volume dissolution vessels, with the coated side exposed to 100 mL SBF. Aliquots of 1.5 ± 0.1 mL were removed at predetermined times up to 144 h and immediately replaced with an equal volume of fresh medium at the same temperature. Samples were filtered through a 0.45 μm RC syringe filter (Phenomenex Inc., Aschaffenburg, Germany), and drug concentrations were determined by HPLC. All analyses were performed in triplicate.

The quantitative analysis of vancomycin was performed via HPLC [55], using a Jasco LC-4000 Series HPLC system (JASCO Corporation, Tokyo, Japan) comprising a PU-4180 quaternary pump, CO-4061 column thermostat, a AS-4150 autosampler and MD-4010 diode array detector. The separation was conducted on a Kinetex EVO C18 Core Shell column with 2.6 µm particle size (Phenomenex Inc., Torrance, CA, USA), maintained at 45 °C. A 10 µL injection volume was used. The mobile phase, delivered at a flow rate of 0.7 mL/min, consisted of 0.1% formic acid and acetonitrile eluted in 88:12 (*v*/*v*) ratio. Quantification was performed at 280 nm.

## 3. Results and Discussion

### 3.1. FT-IR Spectra

**Figure 1:**
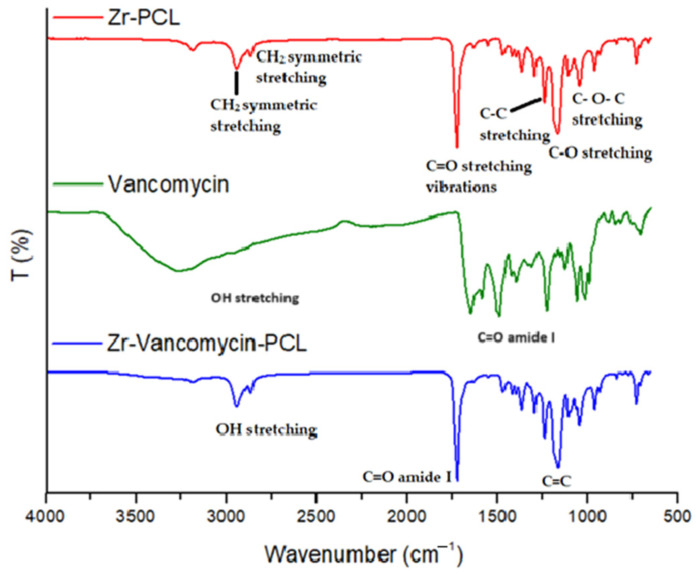
FTIR spectra of Zr-PCL, vancomycin, Zr-vancomycin-PCL on the domain between 500 and 4000 cm^−1^.

**Figure 2:**
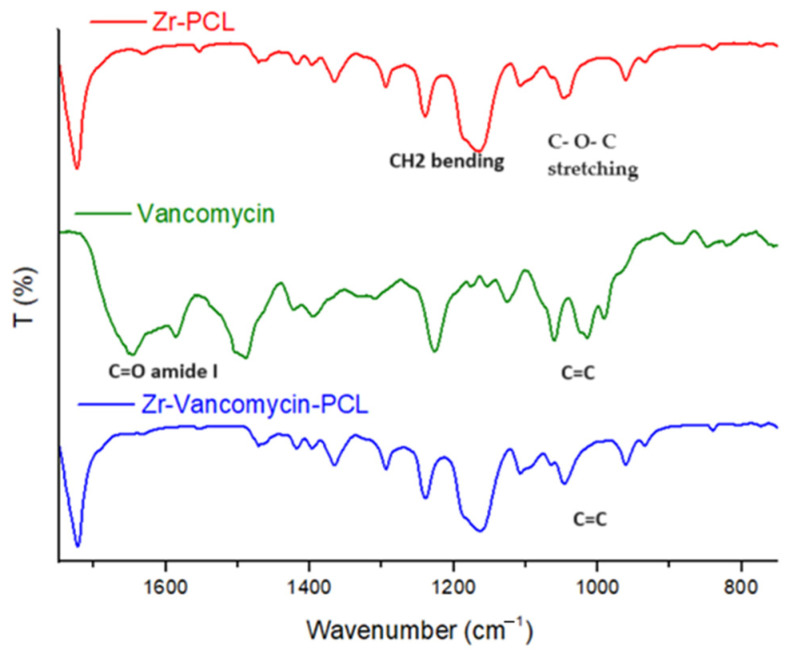
FTIR spectra of Zr-PCL, vancomycin, Zr-vancomycin-PCL, from 750 to 1750 cm^−1^.

Structural functional groups of PCL, electrospun PCL on Zr surface (Zr-PCL), as well as electrospun PCL with vancomycin coatings on Zr samples (Zr-Vancomycin-PCL) were evidenced via FT-IR analysis [56,57] (Figure 1 and Figure 2, and Table 1).

PCL spectra display characteristic peaks of C=O stretching vibrations at 1726 cm^−1^, CH_2_ bending modes at 1361, 1397 and 1473 cm^−1^; CH_2_ asymmetric stretching at 2942 cm^−1^; and symmetric stretching at 2862 cm^−1^. The C–O–C stretching vibrations yield peaks at 1042, 1107 and 1233 cm^−1^. The bands at 1160 and 1290 cm^−1^ are assigned to C–O and C–C stretching [58].

The characteristic peaks of vancomycin include those at 3260–3300 cm^−1^ attributed to hydroxyl stretching, 1651 cm^−1^ corresponding to the C=O amide I and 1050 cm^−1^ associated with the C=C. Bands due to PCL showed that the Zr samples were completely coated with PCL nanowires with characteristic peaks at 1724 cm^−1^, corresponding to C=O; the peaks at 2945 cm^−1^ and 2867 cm^−1^ are attributed to CH_2_. The spectrum of Zr-vancomycin-PCL exhibited the peaks of PCL wires with small peaks of the drug but shifted or with low intensity.

### 3.2. Sample Morphology

**Figure 3:**
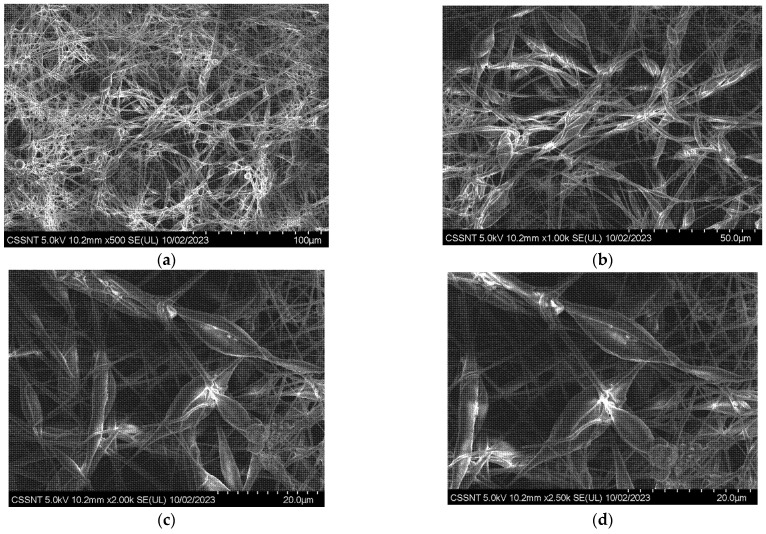
SEM morphologies for Zr-PCL obtained by electrospinning procedure at different magnifications (**a**) ×500; (**b**) ×1 k; (**c**) ×2 k; (**d**) ×2.5 k.

Representative SEM micrographs of Zr covered by electrospun PCL are presented in Figure 3. The surface of the Zr alloy is entirely covered with PCL wires that look like a dense net with granular particles bonded with a linear polymeric chain. At higher magnification, the polymeric chain looks like flower petals bonded with wires (Figure 3b).

**Figure 4:**
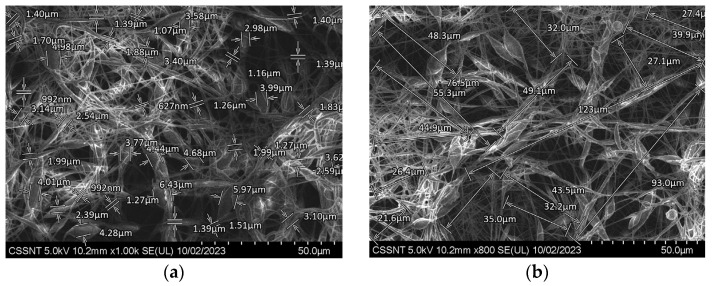
Evaluation of length and thickness on SEM morphologies for Zr covered with PCL by electrospinning procedure at two different magnifications (**a**) ×1 k; (**b**) ×800.

The entire polymer net resembles the petals of a flower joined together by narrow linear zones. To measure the dimensions of these petaloid formations, a quantitative analysis of the SEM micrographs was performed using a function of the SEM software, named SDS (Figure 4a,b).

**Figure 5:**
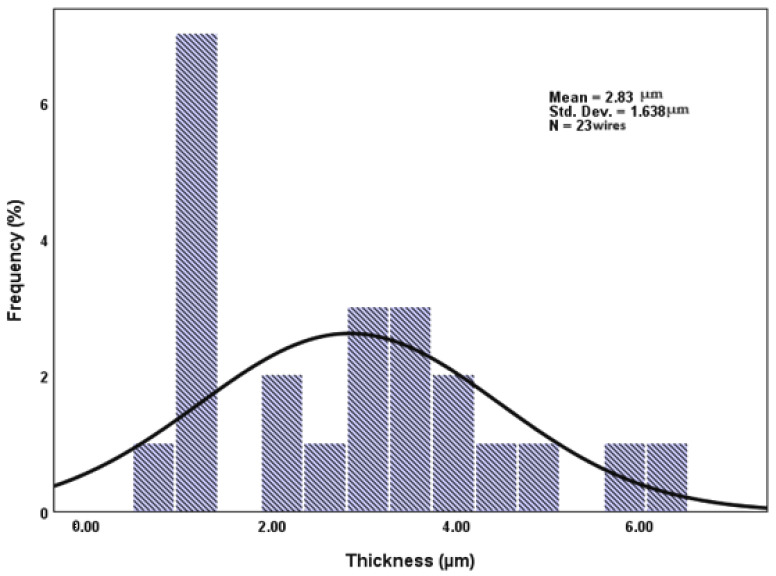
A statistical analysis of the frequency distribution for thickness of PCL wires (SPSS 26 statistical analysis with average value = 2.83 µm, std. dev. = 1.638 µm; N = 23 wires).

**Figure 6:**
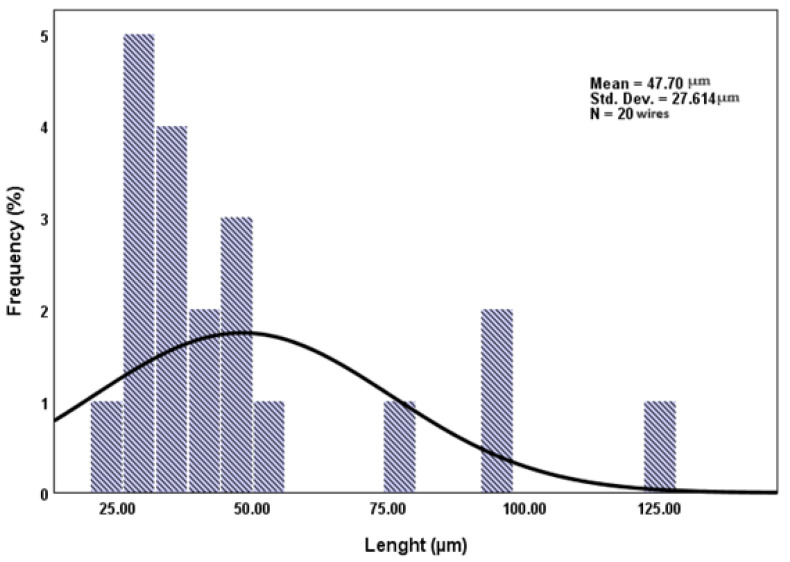
A statistical analysis of the frequency distribution for length of PCL wires (SPSS 26 statistical analysis with average value = 47.70 µm, std. dev. = 27.614 µm; N = 20 wires).

For all the images recorded at higher magnification, a statistical analysis, using SPSS26, was performed, using at least 20 particles measured in triplicate. The results were represented as histograms with their standard deviation (Figure 5 and Figure 6).

The thickness of the wires varies between 627 nm (corresponding to the simple PCL wires) and 6.43 µm (belonging to petaloid formations) (Figure 4a), and the length of the wires varies between 22 and 123 µm (Figure 4b). It should be noted that most wire lengths are normally distributed around 30 µm, with only a small number being longer than 50 µm.

**Figure 7:**
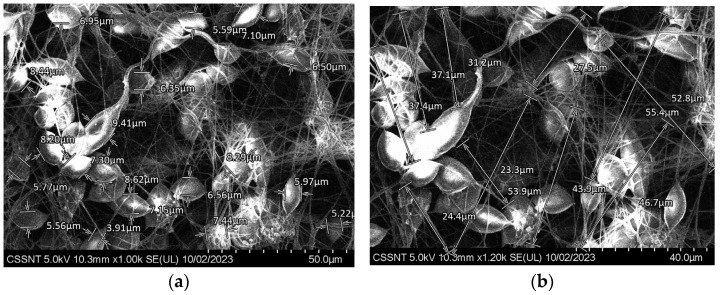
SEM morphologies for Zr covered with PCL and vancomycin by electrospinning procedure at different magnifications (**a**) ×1 k; (**b**) ×1.2 k.

SEM micrographs of Zr-vancomycin-PCL samples are presented in Figure 7a,b.

As can be seen from the SEM micrographs, like the sample covered only with PCL, the sample in which vancomycin was added shows the same petaloid morphology, and no significant difference between Zr-PCL and Zr-vancomycin-PCL appears. The surface of the sample that contains vancomycin is homogenous, covered with wires like a dense net. The thickness of the PCL wires containing vancomycin, measured with SDS-SEM function, on at least 20 wires is between 600 nm and 8.44 µm, and the length is roughly the same as in the Zr-PCL sample.

**Figure 8:**
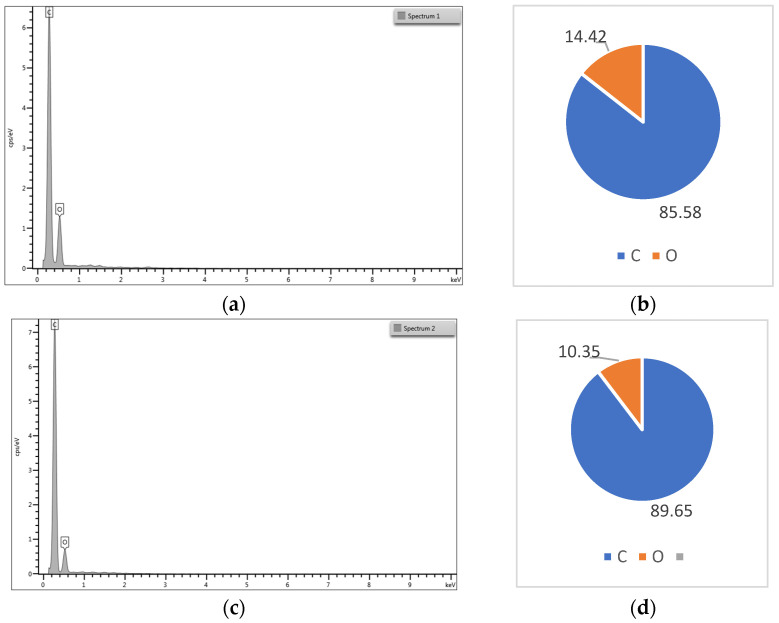
(**a**) Energy dispersive spectra for Zr-PCL; (**b**) elemental distribution in atomic percent for Zr-PCL; (**c**) energy-dispersive spectra for Zr-vancomycin-PCL; (**d**) elemental distribution in atomic percent for Zr-vancomycin-PCL.

The atomic percent for the elements of the PCL and PCL-vancomycin sample is illustrated in Figure 8b,d. From the energy-dispersive analysis (EDS-Figure 8a–d), the presence of vancomycin is probably highlighted by the increasing C atomic % in the quantitative analysis, with almost 5% compared with the sample without vancomycin (Figure 8d compared with Figure 8b).

Even if the intensity of the C peak slowly increases in the sample that contains vancomycin, the increase is small because the vancomycin quantity in the final sample is low.

**Figure 9:**
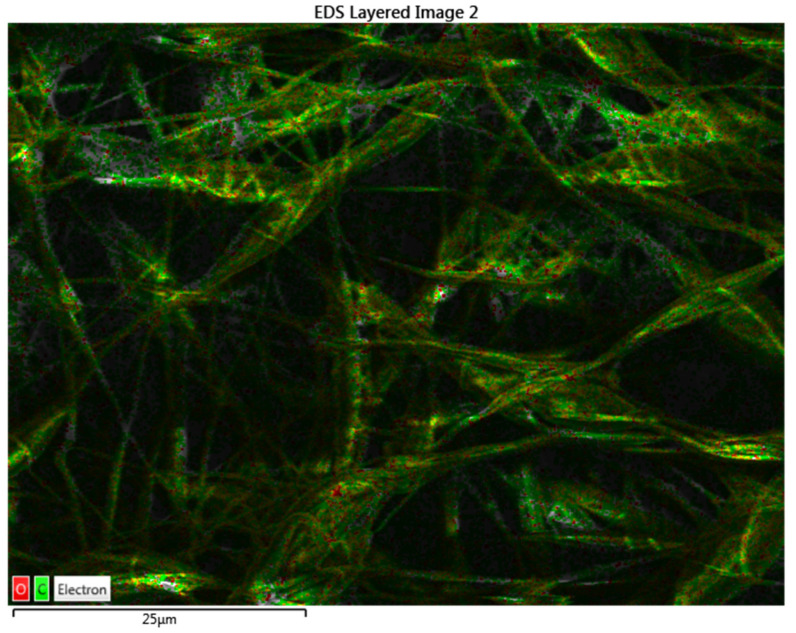

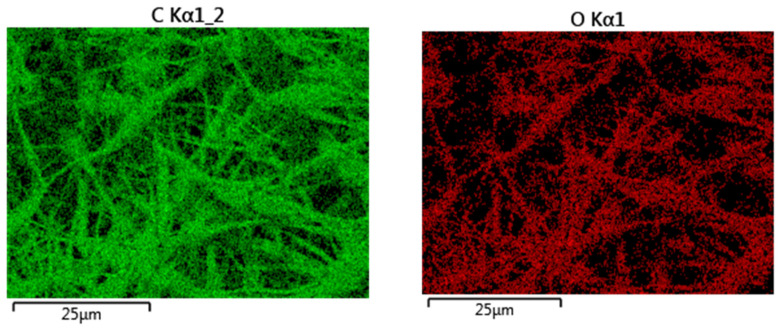
Mapping analysis with elements distribution for Zr-PCL (green-C; red-O).

**Figure 10:**
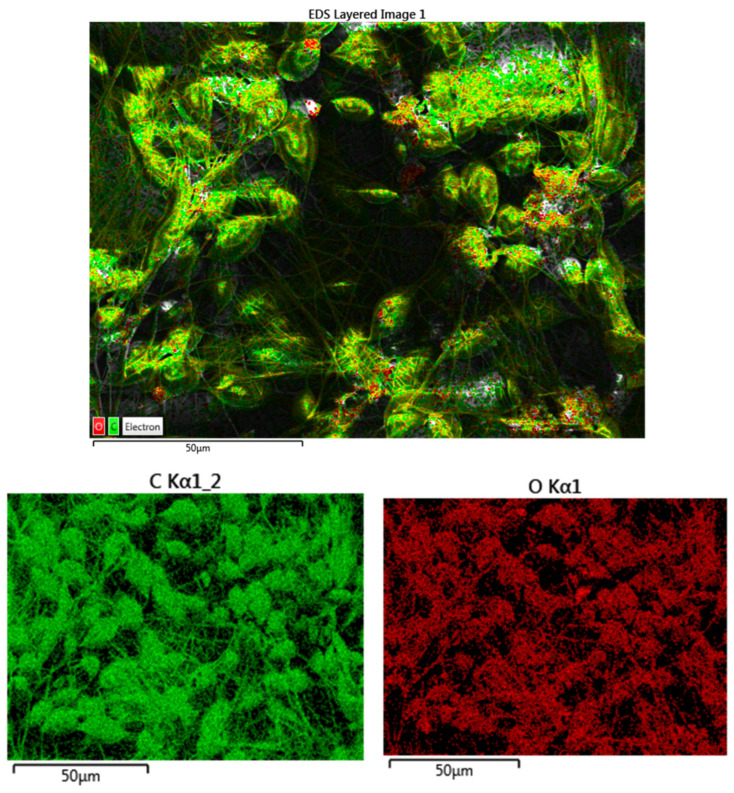
Mapping analysis with elements distribution for Zr-vancomycin-PCL (green-C; red-O).

Using EDS, not only were the spectra and the quantitative analysis obtained but also an elemental map for the Zr-PCL and for Zr-vancomycin-PCL samples. This is an important tool to see the distribution of the elements on the entire surface (Figure 9 and Figure 10).

All the results obtained indicate a uniform distribution of fibers containing C and O on the entire surface of the Zr.

### 3.3. Contact Angle Measurements

**Figure 11:**
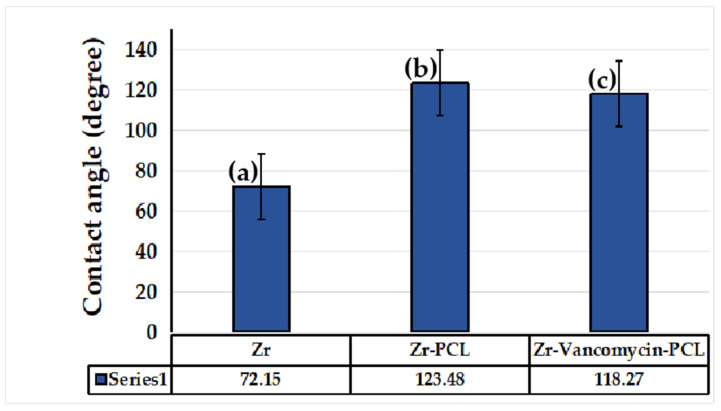
Contact angle measurements for Zr sample (a) uncoated, (b) coated with PCL nanofibers, (c) coated with PCL nanofibers and vancomycin.

The contact angle was performed for five identical samples to assess the hydrophilic or hydrophobic character of the uncoated Zr and coated samples. According to Figure 11, the contact angle for the Zr sample coated with PCL nanofibers increases significantly (123.48°) compared to the uncoated Zr sample (72.15°). As we can see, the Zr-PCL crosses the hydrophobicity limit due to the porous structure of the nanofibers. By embedding vancomycin into PCL nanowires, the contact angle slowly decreases from 123.48° to 118.27°. The contact angle decrease is associated with vancomycin’s ability to form hydrogen bonds due to the fact that the characteristic peak of the hydroxyl stretching is well visible at 3260–3300 cm^−1^ being available to form bonds.

### 3.4. Microhardness Tests

**Figure 12:**
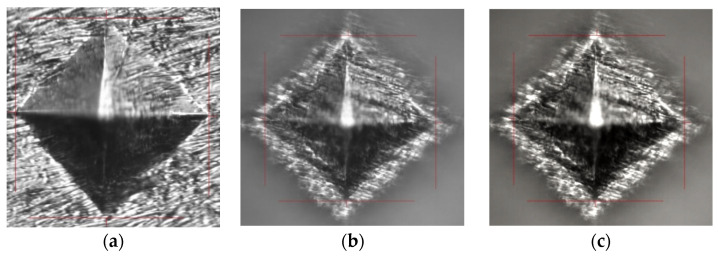
Morphology of samples after microhardness tests (**a**) for Zr sample, (**b**) Zr covered with PCL fibers and (**c**) Zr-vancomycin-PCL.

Vickers microhardness was performed on both the uncoated and coated samples with PCL fiber Zr surfaces to evaluate sample hardness (Figure 12). As we can see in Figure 12a, the indenter trace is visible and shows no cracks on the Zr substrate. In the case of Zr-PCL fibers (Figure 12b), the metallic substrate can be observed, which indicates a thin PCL coating and a vancomycin-PCL coating (Figure 12c). Table 2 shows the microhardness values and the fact that the uncoated Zr surface is harder compared to Zr-PCL fibers and with Zr-vancomycin-PCL. No significant difference between the microhardness of Zr-PCL and Zr-vancomycin-PCL samples appears.

The yield strength was calculated using Tabor’s relationship (2) [59], showing the correlation between the microhardness values and yield strength, as given by:

Yield strength (MPa) = Hardness (MPa)/3 = 9.81 × Hardness in HV/3(2)

### 3.5. Corrosion Resistance Tests (Tafel Plots) and EIS Studies

**Figure 13:**
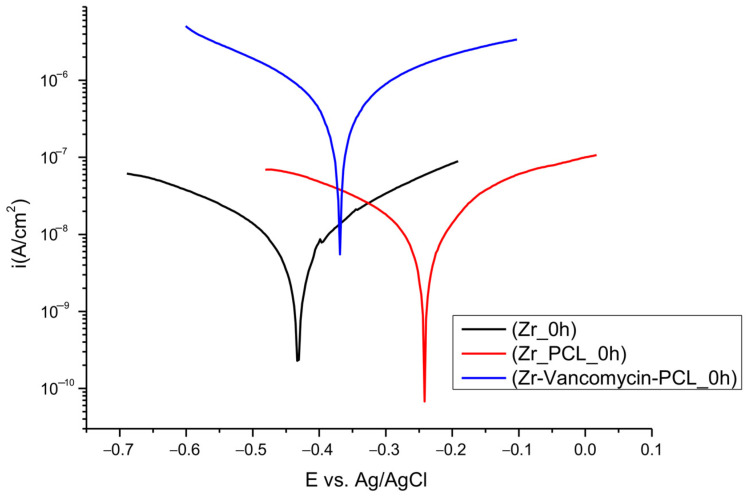
Tafel plots for uncoated and coated Zr with PCL fibers in SBF solution.

Potentiodynamic polarization curves (Figure 13) were established to determine the corrosion resistance of uncoated and coated PCL nanofibers Zr in SBF solution.

The corrosion parameters are illustrated in Table 3 for Zr, Zr-PCL and Zr-vancomycin-PCL, after immersion in SBF, calculated using the Tafel slope extrapolation method.

As can been observed from Table 3, we recorded the corrosion parameters for all samples: E_corr_ = corrosion potential; i_corr_ = corrosion current density; CR = corrosion rate; R_p_ = polarization resistance; ba = anodic Tafel slopes; bc = cathodic Tafel slopes.

While the deposition of PCL occurs, the values of the corrosion rate slightly decrease, and the corrosion potential moves to lower electronegative values.

The highest value for corrosion current density is for Zr-vancomycin-PCL sample, 471.9·10^−9^ A/cm^2^. The lowest polarization resistance is 0.10 × 10^6^ Ω, smaller than for the Zr and Zr-PCL samples. The smallest corrosion rate is for Zr-PCL, 0.00039 mm/year.

All the results indicate that the coatings with PCL have a small positive influence against corrosion at the immersion in a simulated body fluid.

**Figure 14:**
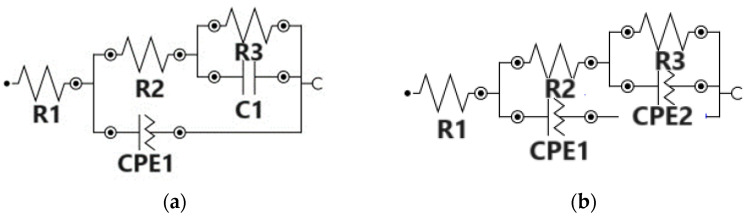
The equivalent circuit for (**a**) Zr; and for (**b**) Zr-PCL and Zr-vancomycin-PCL after immersion in SBF.

**Figure 15:**
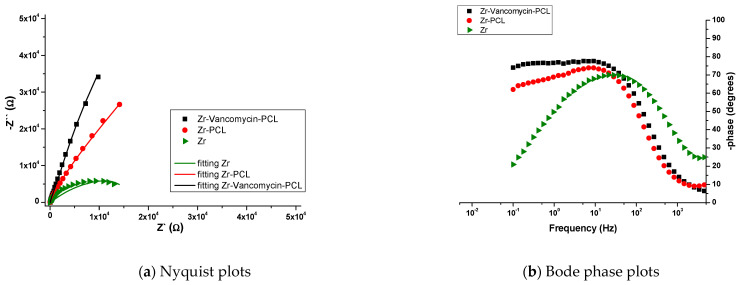
Nyquist (**a**) and Bode (**b**) diagrams for uncoated and PCL- or PCL–vancomycin-coated Zr in SBF.

The impedance measurements were performed at the OCP, with an AC potential amplitude of 10 mV and a frequency range of 100 kHz to 50 mHz. The results obtained from the EIS analysis were fitted using equivalent circuits in NOVA 1.11., the Autolab software NOVA 1.11 (Figure 14), and the EIS parameters are presented in Table 4. Figure 15 presents the Nyquist and Bode diagram recorded for all the samples.

The EIS measurements showed the interfacial change transfer between the electrode and electrolyte, being suggestive of conductance in the samples. Every modification of the EIS parameters shows changes in the chemical processes that occur at the interface metal/solution. By fitting the EIS data, the equivalent circuits were obtained for Zr, Zr-PCL and Zr-vancomycin-PCL samples (Figure 14). Comparing the results, the same circuit is fitted for Zr-PCL and Zr- vancomycin-PCL samples. For all the samples, R1 represents the resistance of the SBF solution. For uncoated Zr, at the interface between Zr and SBF, the elements CPE1 in parallel with R2 represent the possibility that, after immersion in SBF, a thin oxide film appears spontaneously on the surface of Zr. C1 in parallel with resistance R3 represents the double-layer capacitance and, respectively, the charge transfer resistance attributed to the contact between the electrolyte and the substrate. In the case of the PCL and vancomycin-PCL-coated samples, a second interface appears (CPE1 in parallel with R2), representing the coating capacity and coating resistance at the coating−electrolyte interface. At the second interface, R3 (Zr resistance) is in parallel with the second CPE. CPE2 appears in the circuit due to the adsorption–desorption phenomena that occur at the interface between Zr with SBF solution through PCL and vancomycin-PCL coatings. As we observe from Nyquist plots (Figure 15a), a capacitive loop is recorded for uncovered Zr. By comparing Zr-PCL and Zr-vancomycin-PCL, we observe that the loading of the drug does not produce significative changes on the conductive behavior of all samples. In the Bode phase plots (Figure 15b), two capacitive loops appear in the cases of Zr-PCL and Zr-vancomycin-PCL, hinting at the existence of two different layers on the surface of the samples, namely the Zr oxide layer and the polymeric coating. From the Bode plots (Figure 15b), we measure the value of the phase angle, for all the studied samples being between 70 and 80°. The maximum phase angle is obtained for the Zr-vancomycin-PCL sample, almost 80°, indicating a capacitive behavior of the interface. Performing the electrochemical characterization, it can be concluded that from an electrochemical point of view, higher values for coating strength are obtained for the PCL coating, indicating that it provides the best corrosion protection in the SBF electrolyte.

### 3.6. Evaluation of Drug Loading

Vancomycin, as a drug, was loaded on the Zr-PCL fibers. The loading efficiency (LE%) was calculated using a calibration curve. The equation corresponding to the calibration curve is as follows: Abs: y = 0.00588 + 0.00428X, with a correlation coefficient R^2^ = 0.9992. The concentration of vancomycin in the SBF solution was 2 g/L.

**Figure 16:**
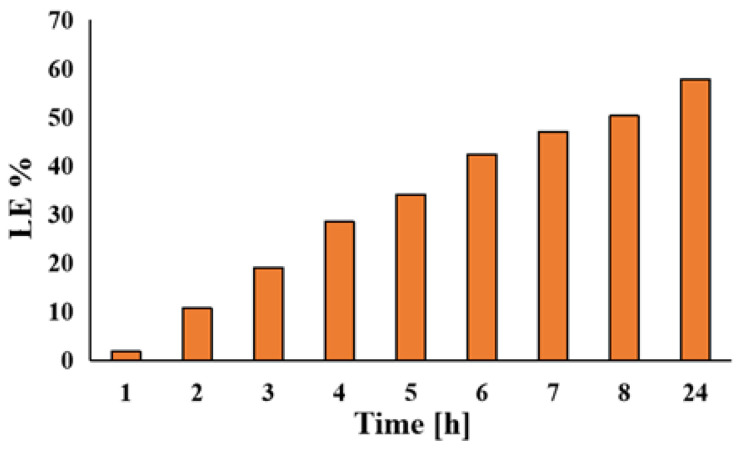
Loading efficiency of vancomycin in SBF solution during 24 h.

The loading percentage of vancomycin was 57.79% (corresponding to 11,558.6 μg/mL) in 24 h (Figure 16).

### 3.7. Evaluation of Drug Release Profile

The release profile of vancomycin loaded into PCL wires was investigated in SBF solution and expressed as a percentage of the total vancomycin loaded.

**Figure 17:**
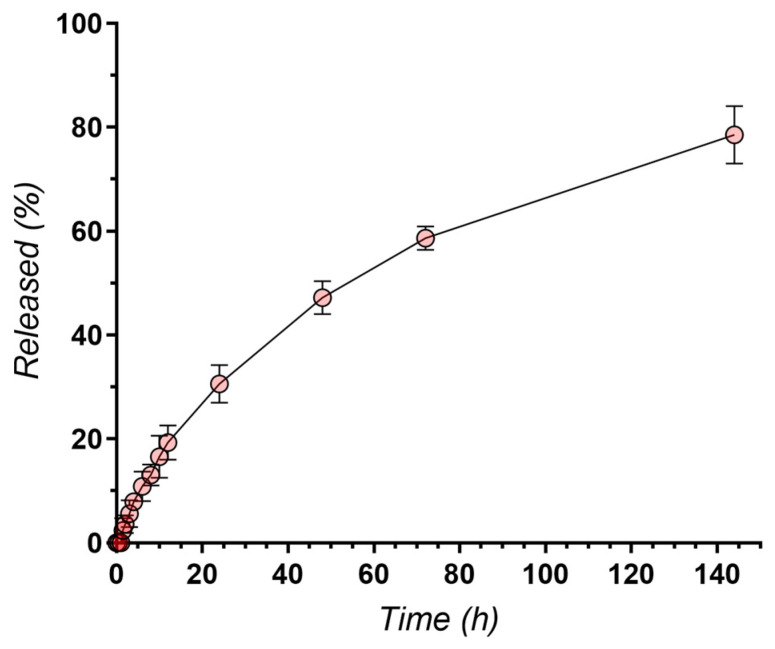
Release profile of vancomycin (n = 3).

The mean release profile of vancomycin from Zr-PCL-vancomycin samples is indicative of a sustained release of the active component, with 78.55% of the total loaded vancomycin being released within 144 h (Figure 17).

It is always critical to ensure that the coating provides sustained release of the antibiotic for the required duration and that it maintains therapeutic drug levels to effectively combat potential bacterial infections [60].

The Zr-PCL-vancomycin samples were able to provide the sustained release of vancomycin for over 7 days, with approximately 80% of the total vancomycin released in 144 h as presented in Supplementary Material. The initial burst release of approximately 30% in the first 24 h is desirable, since an immediate high concentration of the antibiotic is required to combat existing infections and to decrease bacterial adhesion on the surface without compromising biocompatibility. Due to the drug being deposed on the surface rather than embedded in the inner structure of the nanowires, the release rate of vancomycin is higher than that of other polymeric scaffolds [61] but comparable to the release from biodegradable porous polyurethane scaffolds [62], with injectable hydrogels [60] and even polycaprolactone nanofibers [37].

It is also important to note that, after the initial burst, a steady release of vancomycin over 7 days is achieved, which is ideal for prophylactic applications, where the goal is to maintain antibiotic therapeutic levels and to prevent potential infections over a prolonged period.

**Figure 18:**
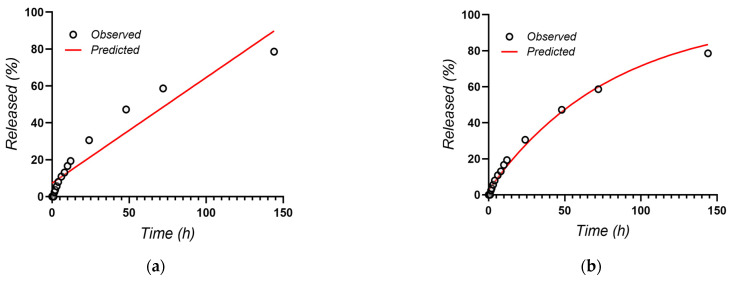

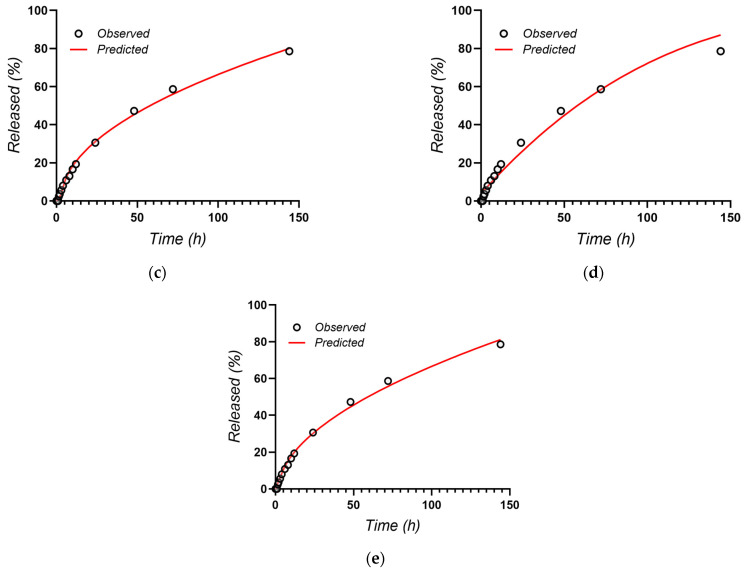
Kinetic models of vancomycin release: (**a**) Zero Order; (**b**) First Order; (**c**) Higuchi; (**d**) Hixson–Crowell; (**e**) Korsmeyer–Peppas.

The release data of vancomycin were fitted using the equations of several diffusion-based kinetic models, such as Zero Order, First Order, Higuchi, Hixson–Crowell and Korsmeyer–Peppas [63] (Figure 18).

The parameters of the equations for the release profiles of vancomycin are presented in Table 5.

The results showed that the Korsmeyer–Peppas model was characterized by the highest regression coefficient (R^2^ = 0.9938) and was the best fitting model of vancomycin release from PCL nanowires, whereas the value of the release exponent was very close to 0.5 (n = 0.539), suggesting the drug release as mostly a diffusion process based on Fick’s law (square root time dependent (as also suggested by the very good fitting of the Higuchi model)). However, the slightly higher than 0.5 value of the n exponent in the Korsmeyer–Peppas model also suggests a coupling of the diffusion, with a slight erosion/relaxation-based release mechanism.

Another recent paper, ref. [64], affirmed that, even over longer times, vancomycin has a release effect, and no toxicity was observed.

As SEM images confirm, the surface of Zr coated with nanofibers has a uniform coverage. According to the drug loading value after 24 h, which denotes permeability as well, the kinetic model sustains drug transport with a Fick diffusion part and a relaxation part, including both adsorption and desorption processes.

## 4. Conclusions

Using the electrospinning method, PCL fibers were successfully deposited onto the Zr sample. Vancomycin was added into PCL fibers, and the deposition was studied using FT-IR, SEM-EDS, contact angle, microhardness and two electrochemical methods. The dimensions of the polymeric wires were analyzed using a function of SEM software (SDS). The EDS emphasized the small increase in the C% of vancomycin by the increase in the C intensity peak in the spectra of the Zr-vancomycin-PCL sample.

The morphologies of the PCL and PCL-vancomycin wires appear to be similar. No significant differences were highlighted. The length of the wires was up to 300 µm.

The CA for the electrospun samples shows a very slight hydrophobic behavior because PCL is a polymer. By deposition of PCL and PCL-vancomycin fibers, the microhardness decreases due to the poor mechanical properties of the polymer. The coatings with PCL present a positive influence against corrosion after immersion in SBF. From the EIS studies, the sample containing vancomycin has almost the same behavior as Zr-PCL, the sample covered with PCL being more stable to corrosion than uncovered Zr.

The loading percentage of vancomycin was 57.79% in 24 h. The release of the drug from the PCL fibers was studied over 144 h, and the kinetic study was performed using five mathematical models. The Korsmeyer–Peppas model based on Fickian diffusion was found to best describe the vancomycin release kinetics from PCL nanowires.

## Figures and Tables

**Table 1:** FT-IR specific assignment for PCL and for vancomycin peaks from the literature [37,58].

Sample	Wave Number (cm^−1^)	Characteristic Peaks
PCL	1726	C=O stretching vibrations
	1361, 1397 and 1473 cm^−1^	CH_2_ bending modes
	2942	CH_2_ asymmetric stretching
	2862	CH_2_ symmetric stretching
	1042, 1107 and 1233	C–O–C stretching vibrations
	1160 and 1290	C–O and C–C stretching
Vancomycin	3422	N-H band
	3260–3300	OH stretching
	1651	C=O amide I
	1413	C–N band
	1050	C=C

**Table 2:** Values of Vickers microhardness of the Zr samples.

Sample	Vickers Hardness (HV)	Yield Strength (MPa)
Zr	9.66	31.58
Zr-PCL	7.33	23.96
Zr-Vancomycin-PCL	7.21	23.57

**Table 3:** Corrosion parameters of the samples.

Samples	E_corr_ (V)	i_corr_ (A/cm^2^)	CR (mm/Year)	R_p_ (Ω)	ba (V/dec)	bc (V/dec)
Zr	−0.421	8.66·10^−9^	0.00040	5.89·10^6^	0.278	0.203
Zr-PCL	−0.232	8.48·10^−9^	0.00039	3.91·10^6^	0.199	0.124
Zr-Vancomycin-PCL	−0.365	471.9·10^−9^	0.02885	0.10·10^6^	0.217	0.223

**Table 4:** Equivalent circuit values for Zr; Zr-PCL and Zr- vancomycin-PCL samples.

Sample	R1 [Ω]	R2 [kΩ]	CPE1		R3 [kΩ]	CPE2		C [F]
			Yo [Mho]	N		Yo [Mho]	N	
Zr	27.6	12.2	29.4·10^−6^	0.827	4.47			1.27·10^−4^
Zr-PCL	68.8	42.4	18.7·10^−6^	0.951	1100	30.3·10^−6^	0.563	-
Zr-PCL-Vancomycin	41	28	34.9·10^−6^	0.903	386	8.1·10^−6^	0.862	-

**Table 5:** Vancomycin release parameters of the five kinetics models.

Sample	Zero Order		First Order		Higuchi		Hixson-Crowell		*Korsmeyer-Peppas*		
	R^2^	k_0_	R^2^	k_1_	R^2^	K_H_	R^2^	k_3_	R^2^	k_KP_	n
Zr-PCL-Vancomycin	0.900	0.573	0.9873	0.012	0.9934	6.725	0.9713	0.003	***0.9938***	***5.749***	***0.534***

## Data Availability

Data is contained within the article.

## Supplementary Materials

The following supporting information can be downloaded at: https://www.mdpi.com/article/10.3390/ma16227237/s1. Figure S1. Representative chromatogram obtained for the release of Vancomycin from Zr-PCL-Vancomycin sample (time = 144 h).
