# Supplementary material for: Electrospun PCL Wires Loaded with Vancomycin on Zirconium Substrate
Source: Materials (Basel). 2023 Nov 20;16(22):7237. doi: 10.3390/ma16227237 (PMC10672849; doi:10.3390/ma16227237)
Supplement: Supplementary file 1 [file materials-16-07237-s001.zip › materials-2722856-supplementary.pdf]

materials

Supplementary material

Electrospun PCL Wires Loaded with Vancomycin on Zirconium Substrate

Ramona-Daniela Radu (Dusman) [1], Manuela Elena Voicu [1], Mariana Prodana [1,*], Ioana Demetrescu [1,2], Valentina Anuta [3] and Doina Draganescu [4]

Quantification of Vancomycin release

A representative chromatogram obtained for the quantification of the Vancomycin released from the Zr-PCL-Vancomycin samples is presented in Figure S1.

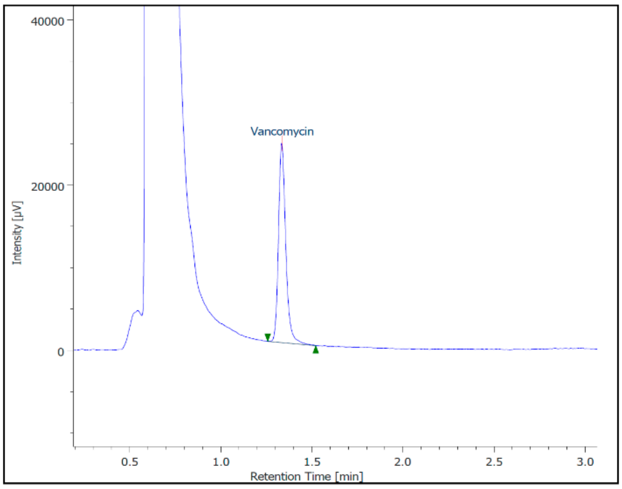

Figure S1. Representative chromatogram obtained for the release of Vancomycin from Zr-PCL-Vancomycin sample (time= 144 hours).
